# Distinct prophase arrest mechanisms in human male meiosis

**DOI:** 10.1242/dev.160614

**Published:** 2018-04-16

**Authors:** Sabrina Z. Jan, Aldo Jongejan, Cindy M. Korver, Saskia K. M. van Daalen, Ans M. M. van Pelt, Sjoerd Repping, Geert Hamer

**Affiliations:** 1 Center for Reproductive Medicine, Amsterdam Research Institute Reproduction and Development, Academic Medical Center, University of Amsterdam, 1105 AZ, Amsterdam, The Netherlands; 2 Bioinformatics Laboratory, Department of Clinical Epidemiology, Biostatistics and Bioinformatics, Academic Medical Center, 1105 AZ, Amsterdam, The Netherlands

**Keywords:** Human spermatogenesis, Infertility, Meiosis, Meiotic arrest, Meiotic silencing, p63

## Abstract

To prevent chromosomal aberrations being transmitted to the offspring, strict meiotic checkpoints are in place to remove aberrant spermatocytes. However, in about 1% of males these checkpoints cause complete meiotic arrest leading to azoospermia and subsequent infertility. Here, we unravel two clearly distinct meiotic arrest mechanisms that occur during prophase of human male meiosis. Type I arrested spermatocytes display severe asynapsis of the homologous chromosomes, disturbed XY-body formation and increased expression of the Y chromosome-encoded gene *ZFY* and seem to activate a DNA damage pathway leading to induction of p63, possibly causing spermatocyte apoptosis. Type II arrested spermatocytes display normal chromosome synapsis, normal XY-body morphology and meiotic crossover formation but have a lowered expression of several cell cycle regulating genes and fail to silence the X chromosome-encoded gene *ZFX*. Discovery and understanding of these meiotic arrest mechanisms increases our knowledge of how genomic stability is guarded during human germ cell development.

## Abstract (precis)

**Summary:** Histological examination and transcriptomic analysis of human meiosis-arrested spermatocytes reveals two prophase arrest mechanisms, each associated with distinct gene expression profiles implicating the involvement of different biological processes.

## INTRODUCTION

Whereas our somatic bodies inevitably die of old age or disease, our germ cells have to maintain sufficient genomic integrity to pass on our genome to, in principle, endless generations. Therefore, to prevent transmission of aneuploidies or other chromosomal aberrations, strict genome integrity checkpoints exist in the process of meiosis to remove germ cells that fail certain quality checks.

During meiosis, in order to generate haploid sperm or oocytes, diploid germ cells undergo two consecutive rounds of chromosome segregation after one round of DNA replication. During meiosis I, the homologous chromosomes, each consisting of one pair of sister chromatids, are segregated, followed by separation of the sister chromatids into haploid cells during meiosis II. Successful meiosis requires that the homologous chromosomes are properly paired and aligned. This is achieved by the induction of DNA double-strand breaks (DSBs) by the protein SPO11 during the prophase of the first meiotic division. The repair of these SPO11-induced DSBs initiates and requires synapsis of the homologous chromosomes and ensures the formation of at least one meiotic crossover per homologous chromosome pair (De Massy, 2013).

In the mouse, failure to repair meiotic DSBs properly or synapse the homologous chromosomes leads to arrest during the first meiotic prophase at a specific stage of spermatogenesis, termed epithelial stage IV arrest (De Rooij and De Boer, 2003; Hamer et al., 2008). However, despite displaying spermatocyte apoptosis at the same stage of spermatogenesis, different meiotic recombination mouse mutants show different responses and cytological end-points (Barchi et al., 2005). This led to the idea that more than one checkpoint mechanism exists that can induce apoptosis of meiotic cells at stage IV of mouse spermatogenesis.

One type of mouse stage IV arrest occurs independently of SPO11-induced DSBs (Baudat et al., 2000; Romanienko and Camerini-Otero, 2000) or the conventional DNA damage response protein p53 (TRP53) (Odorisio et al., 1998; Yuan et al., 2001), and is caused by incomplete synapsis of the homologous chromosomes (Burgoyne et al., 2009; Jan et al., 2012). When homologous chromosomes synapse, the checkpoint protein TRIP13 removes the meiosis-specific HORMA domain proteins HORMAD1 and HORMAD2 from the chromosome axes (Wojtasz et al., 2009). However, on asynapsed chromosome axes these proteins remain present and recruit the kinase ATR (Wojtasz et al., 2012; Daniel et al., 2011), which, together with several other proteins such as BRCA1 and γH2AX, mark the silencing of transcription from asynapsed chromosomal regions via a process referred to as meiotic silencing (Royo et al., 2013; Turner, 2015). Usually, when the autosomes are fully synapsed, only the X and Y chromosomes are subject to meiotic silencing because they remain largely unsynapsed owing to a lack of sequence homology. This leads to the formation of the XY body, in which the sex chromosomes are bound to ATR, BRCA1 and γH2AX (Burgoyne et al., 2009; Jan et al., 2012). However, in case of extensive autosomal asynapsis, these proteins are sequestered away from the sex chromosomes leading to failure to silence the Y chromosome. Studies have shown that, in particular, lack of timely silencing of the mouse Y chromosome genes *Zfy1* and *Zfy2* induces apoptosis of spermatocytes at stage IV of spermatogenesis via a yet unknown mechanism (Royo et al., 2010).

Because synapsis of the homologous chromosomes and meiotic recombination are two highly intertwined events, i.e. problems with recombinational repair will usually also lead to asynapsis and vice versa, the possibility that two separate meiotic checkpoints may act at stage IV of spermatogenesis has long been overlooked. However, it has been found that the canonical DNA damage response pathway, consisting of MRE11, NBS1 (NBN), ATM and the checkpoint kinase CHK2 (CHEK2), can induce mouse spermatocyte apoptosis prior to XY-body failure-induced apoptosis (Pacheco et al., 2015). Moreover, in mouse oocytes, in which apoptosis cannot be induced by *Zfy* expression because of the absence of a Y chromosome, unrepaired meiotic DSBs also activate CHK2, which, subsequently, provokes apoptosis via the DNA damage response proteins p53 and p63 (TRP63) (Di Giacomo et al., 2005; Li and Schimenti, 2007; Bolcun-Filas et al., 2014). Indeed, both p53 and p63 are also present in mouse spermatocytes (Beumer et al., 1998; Hamer et al., 2001) and have been recently found to be specifically involved in recombination-dependent pachytene arrest of mouse spermatocytes (Marcet-Ortega et al., 2017).

In contrast to the mouse, the mechanisms of human meiotic arrest have not been thoroughly investigated at the molecular level and are poorly understood. Nevertheless, about 10-20% of men with non-obstructive azoospermia are diagnosed with complete or incomplete meiotic arrest (Su et al., 1999; Tsai et al., 2012). Some of these men are carriers of a known genetic aberration, for instance a defined chromosomal translocation or duplication (Sciurano et al., 2011) or a single gene mutation that has been associated with meiotic arrest (Miyamoto et al., 2003; Röpke et al., 2013; Mou et al., 2013; Yang et al., 2015; Yatsenko et al., 2015), but the etiology remains unknown in the vast majority of men. The hundreds of possible genes or unknown environmental factors that could be involved, and the lack of appropriate human genetic models, has meant that a general meiotic arrest mechanism has not been determined in humans.

## RESULTS

### Two types of human meiotic prophase arrest

**Fig. 1.**
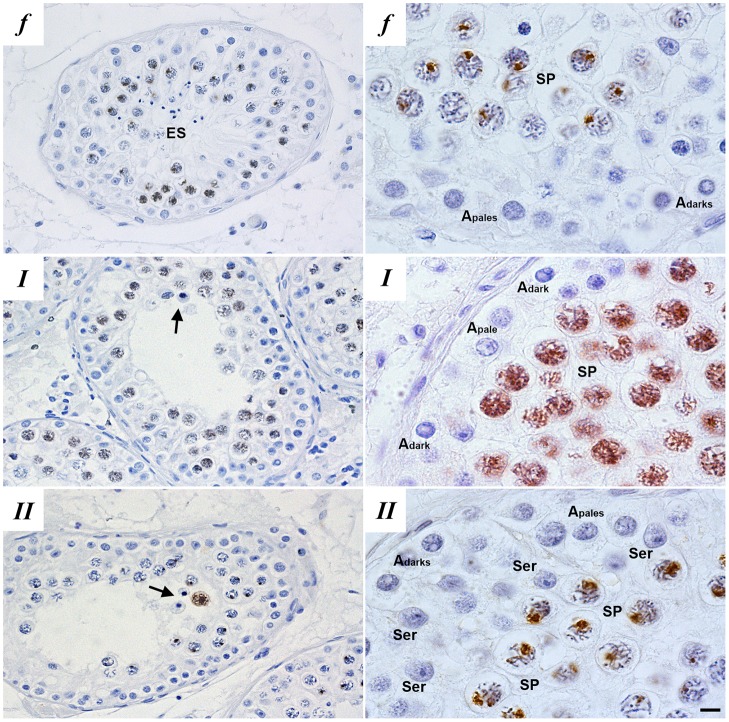
**Histological** **evaluation of patient testis sections.** Immunohistochemical localization of γH2AX in paraffin-embedded testis sections of fertile men (*f*) and patients with meiotic maturation arrest reveal two types of meiotic prophase arrest patients: type I (*I*) and type II (*II*) meiotic arrest. Type I meiotic arrest patients display meiotic prophase arrest and disturbed γH2AX distribution and no XY-body formation, type II meiotic arrest patients display meiotic prophase arrest but normal γH2AX distribution and XY-body formation. Left-hand panels show a global overview of the γH2AX staining in different germ cell populations within the testis and the right-hand panels show higher magnification images of γH2AX staining in spermatocytes. Depicted are: Sertoli cells (Ser), elongated spermatids (ES), A_pale_ and A_dark_ spermatogonia, spermatocytes (SP) and apoptotic spermatocytes (arrows). Scale bars: 3 μm (left); 10 μm (right).

**Fig. 2.**
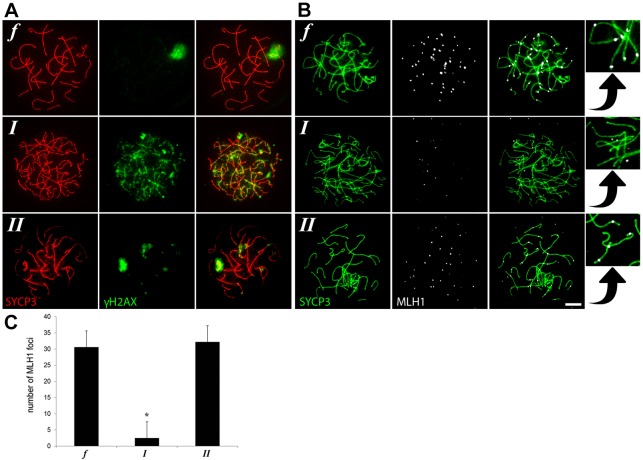
**Immunofluorescence staining of meiotic spread preparations of fertile men (*f*) and type I (*I*) and type II (*II*) meiotic arrest patients.** (A,B) γH2AX (A), MLH1 (labeling meiotic crossovers; B) and SYCP3 (A,B) immunostaining. Scale bar: 5 μm. Insets on the right are a magnification of the neighboring panels showing MLH1 staining in relation to SYCP3. (C) Average number of MHL1 foci (±s.d.) in pachytene spermatocytes of fertile men (*n=*10) and type I (*n=*7) and type II (*n=*10) arrested human spermatocytes. *P≤0.00001 (one-way ANOVA).

To investigate human meiotic arrest, we collected testis biopsies from men with non-obstructive azoospermia diagnosed with maturation arrest. From 2011 to 2013, we collected 350 testicular biopsies, of which 14 displayed complete meiotic arrest. After careful histological examination, four samples were deemed unfit for single-cell laser dissection microscopy (LDM) owing to poor morphology of the testicular sections. The remaining ten patients displayed meiotic arrest at a stage of the seminiferous epithelium that normally contains pachytene spermatocytes. At the histological level, these arrested cells appeared pachytene-like, based on association of the arrested spermatocytes with earlier germ cells in specific stages of the seminiferous epithelium, nuclear morphology and spermatocyte size [explained in detail by Jan et al. (2017), specifically figure 2 from this article]. In these patients, we first investigated whether the arrested spermatocytes had formed a normal XY body. We therefore stained paraffin-embedded testis sections with an antibody against γH2AX (Fig. 1). Before completion of synapsis of the homologous chromosomes at the zygotene stage, γH2AX marks all asynapsed chromosome axes. Subsequently, in healthy pachytene spermatocytes, γH2AX becomes restricted to the XY body, in which the X and Y chromosomes are not fully synapsed and are transcriptionally silenced (Burgoyne et al., 2009; Jan et al., 2012; Turner, 2015). In five men, hereafter referred to as type I, meiotic prophase arrest was characterized by the absence of a discernible XY body and by γH2AX staining dispersed throughout the nucleus. The other five men, hereafter referred to as type II, also displayed meiotic prophase arrest but showed similar γH2AX staining as in controls that exhibited normal spermatogenesis (Fig. 1).

In order to investigate XY-body formation and homologous chromosome synapsis further, we made meiotic spread preparations from biopsies of the same patients to stain for γH2AX and SYCP3, which loads onto meiotic chromosomes prior to synapsis to promote assembly of the synaptonemal complex. In contrast to controls with normal spermatogenesis, the most advanced spermatocytes in type I patients displayed severe asynapsis of the homologous chromosomes, characterized by a zygotene-like appearance of SYCP3, and absence of an XY body, marked by dispersed γH2AX staining covering all asynapsed chromosomes (Fig. 2A, I). The most advanced spermatocytes from type II patients, on the other hand, reached full chromosome synapsis and formed XY bodies similar to controls with normal spermatogenesis (Fig. 2A, II). In the mouse, γH2AX has been shown to mark meiotic silencing of asynapsed chromosomes (Burgoyne et al., 2009; Jan et al., 2012; Turner, 2015). To investigate whether this is also the case in human spermatocytes, we combined γH2AX with an RNA staining protocol for Cot-1 to mark RNA synthesis. Using confocal microscopy and maximum projection of the confocal layers, we found that, also in human spermatocytes, transcriptionally silent regions are marked by γH2AX (Fig. S1). Moreover, we could not find spermatocytes from type I patients displaying γH2AX positive XY bodies normally marking the transcriptionally silent sex chromosomes.

To evaluate meiotic progression further, we used staining against MLH1 to investigate whether meiotic crossover formation is disturbed. As expected, spermatocytes from type I patients never proceeded beyond a zygotene-like stage with asynapsed homologous chromosomes and very little MLH1 staining on the chromosome axes marked by SYCP3 (Fig. 2B, I and insets, Fig. 2C). However, like in the controls with normal spermatogenesis, the most advanced spermatocytes from type II patients seemed more pachytene-like with fully synapsed homologous chromosomes and MLH1 foci on the chromosome axes marked by SYCP3 (Fig. 2B, II and insets, Fig. 2C).

Thus, based on these histological and cytological evaluations of testis samples of men with meiotic arrest from our clinic we identified the existence of two types of human meiotic prophase arrest. One group shows aberrant XY-body formation, severe asynapsis of the homologous chromosomes and meiotic arrest comparable to stage IV arrest in mouse. A second group displays normal chromosome synapsis, XY-body morphology and crossover formation and is distinct from stage IV meiotic arrest in mouse.

### Different types of arrested spermatocytes have distinct gene expression profiles

**Fig. 3.**
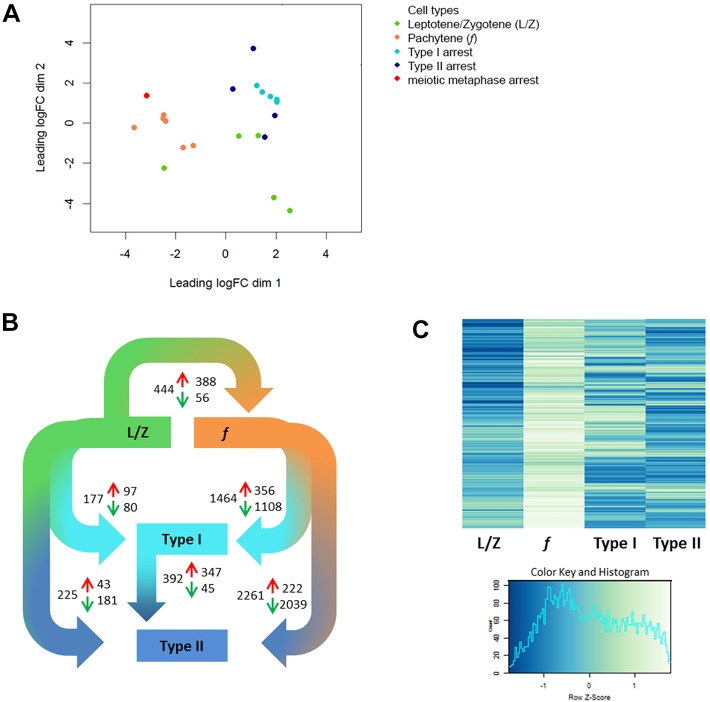
**Transcriptomic analysis of fertile (*f*), type I and II spermatocytes.** (A,B) Multidimensional scaling of leptotene/zygotene spermatocytes (L/Z, green) and pachytene spermatocytes (*f*, orange) from fertile men (data taken from Jan et al., 2017) alongside type I (turquoise), type II (dark blue) and meiotic metaphase (metaphase, red) arrested spermatocytes (A) and differential gene expression analysis (adjusted *P*-value<0.05; B) reveal distinct transcriptomic profiles for type I and II arrested spermatocytes. Depicted in B are comparisons between leptotene/zygotene (L/Z) and pachytene spermatocytes from fertile men; pachytene spermatocytes from fertile men and type I and II arrested spermatocytes; leptotene/zygotene (L/Z) and type I and II arrested spermatocytes; and, finally, between type I and type II arrested spermatocytes. The total number of DEGs for every comparison are shown as well as the number of genes upregulated (red arrows) and the number of genes downregulated (green arrows). (C) Analysis of genes upregulated during the leptotene/zygotene to pachytene transition in normal spermatogenesis reveals a leptotene/zygotene-like expression pattern in type I and II arrested spermatocytes.

In order to try to understand the molecular mechanisms underlying these different types of meiotic arrest, we used a protocol for single-cell LDM and RNA sequencing (Jan et al., 2017) to generate the transcriptomic profiles of arrested spermatocytes from the ten patients with meiotic arrest. From each patient, 500 morphologically normal and non-apoptotic histologically pachytene-like spermatocytes were isolated from tubular cross-sections and pooled for further RNA sequencing and analysis. Comparison of these profiles with expression profiles of leptotene/zygotene spermatocytes and pachytene spermatocytes with similar morphology and from a similar spermatogenic stage, but derived from men with normal spermatogenesis (Jan et al., 2017), revealed that type I and II arrested spermatocytes are transcriptomically distinct from normal spermatocytes. At the transcriptome level, most arrested spermatocytes appeared to be more leptotene/zygotene-like than pachytene-like (Fig. 3A). Notably, type I arrested spermatocytes clustered more closely together, whereas we observed more biological variation between type II arrested spermatocytes (Fig. 3A). Spermatocytes derived from one patient, thus far classified as type II, clustered closely to controls with normal spermatogenesis (Fig. 3A). We then evaluated more testis sections of all patients and found that spermatogenesis of this particular patient progressed beyond the first meiotic prophase and arrested later at a meiotic metaphase stage. This was not immediately visible in the beginning of this study because only very few tubules contain the stage of the seminiferous epithelium that contains meiotic metaphase spermatocytes (Fig. S2). Because only a single patient displayed this type of arrest we decided to exclude this patient from further downstream analysis to avoid drawing too far-reaching conclusions based on a single case.

Analysis of differentially expressed genes (DEGs) confirmed that both type I and II arrested spermatocytes more closely resemble leptotene/zygotene-like cells than normal pachytene spermatocytes (Fig. 3B, Table S1). We therefore looked more closely at the expression of genes that we previously found to be upregulated during the leptotene/zygotene to pachytene transition (Jan et al., 2017). This analysis first revealed that many genes from this gene set were not upregulated in type I or type II arrested spermatocytes but remained at leptotene/zygotene-like expression levels (Fig. 3C). Moreover, the two types of meiotic prophase arrest were clearly distinct, showing different sets of genes that fail to reach normal pachytene expression levels (Fig. 3C).

### The two types of meiotic prophase arrest are characterized by specific biological processes

**Fig. 4.**
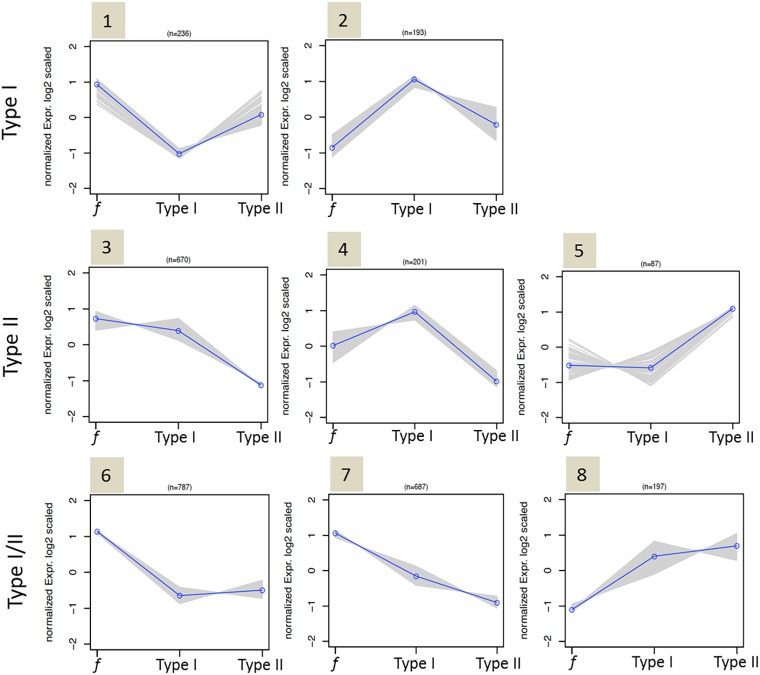
**Clustering of upregulated or downregulated genes in type I, type II (or in both) arrested spermatocytes.** K-means cluster analysis of DEGs between fertile (*f*), type I and type II spermatocytes reveals genes that are aberrantly expressed in type I arrested spermatocytes (clusters 1, 2), type II arrested spermatocytes (clusters 3, 4, 5) and in both type I and II arrested spermatocytes (clusters 6, 7, 8). Blue line represents the mean expression profile of genes in a cluster; profiles of individual genes are depicted by gray lines.

Subsequent k-means clustering of the DEGs, based on their expression profile in fertile, type I or type II arrested spermatocytes, generated eight major gene expression clusters (Fig. 4, Table S2). In order to investigate the differences in molecular pathways between the two patient groups, we performed gene ontology analysis using DAVID on these clusters (Table 1, Table S3).

**Table 1.**
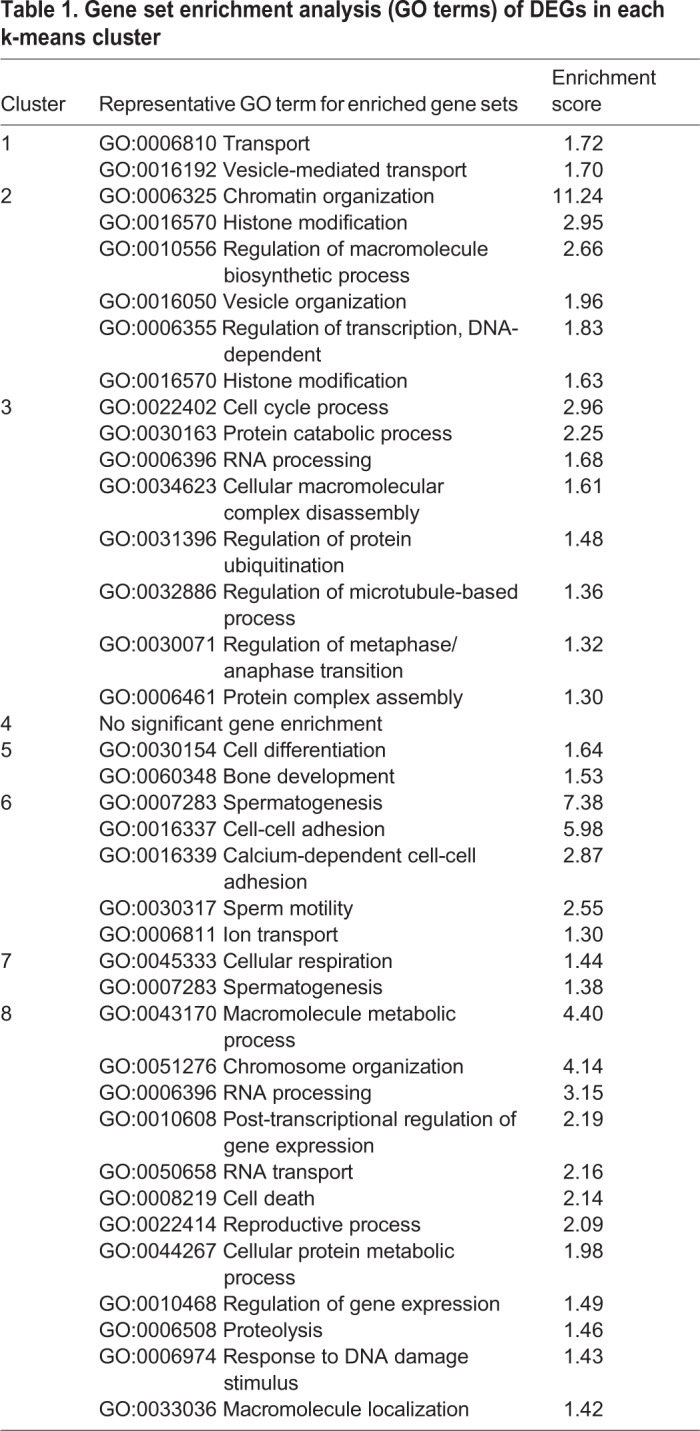
**Gene set enrichment analysis (GO terms) of DEGs in each k-means cluster**

Clusters 1 and 2 consisted of 429 genes that were aberrantly expressed specifically in type I arrested spermatocytes. Of these, 236 genes, predominantly involved in vesicle formation and transport, were downregulated in type I arrested spermatocytes (cluster 1) and 193 genes were specifically upregulated in type I arrested spermatocytes (cluster 2). Reflecting the clear defect in chromosome synapsis in type I arrested spermatocytes, this cluster was highly enriched (enrichment score: 11.24 with >1.3 being significant) with a gene set representing chromatin organization. In addition, genes involved in histone modification were also clearly upregulated, as were genes involved in nucleic acid metabolic processes and gene transcription.

Clusters 3, 4 and 5 consisted of 958 genes that were aberrantly expressed specifically in type II arrested spermatocytes, of which 871 genes were downregulated (clusters 3 and 4). This gene set included many genes involved in cell cycle progression, including the cyclins A1, A2 and E1, as well as genes involved in microtubule organization and the metaphase to anaphase transition. Also, genes involved in macromolecule (protein) degradation and RNA processing were clearly over-represented in these clusters. The remaining 87 genes that were specifically upregulated in type II spermatocytes (cluster 5) were enriched for a gene set involved in cellular differentiation.

Clusters 6, 7 and 8 consisted of 1671 genes that were aberrantly expressed in both type I and II arrested spermatocytes. Of these, 1474 genes were downregulated in both types of arrested spermatocytes (clusters 6 and 7). These genes appeared to be predominantly involved in spermatogenesis, cell-cell adhesion and sperm motility. The remaining 197 genes upregulated in both type I and type II spermatocytes (cluster 8) are involved in numerous processes, including macromolecule metabolic processes (for instance nucleic acid metabolic processes), chromosome structure and organization, RNA processing/transport, cell death and post-transcriptional regulation of gene expression.

In summary, both types of meiotic arrest was associated with upregulation of genes involved in chromatin structure and organization and RNA processing. However, in type I arrested spermatocytes chromatin structure and organization appeared to be the major underlying problem, whereas type II meiotic arrest was characterized by downregulation of genes involved in RNA processing and cell cycle progression.

### Type I and II specific upregulation of the sex chromosome-encoded genes *ZFY* and *ZFX*

**Fig. 5.**
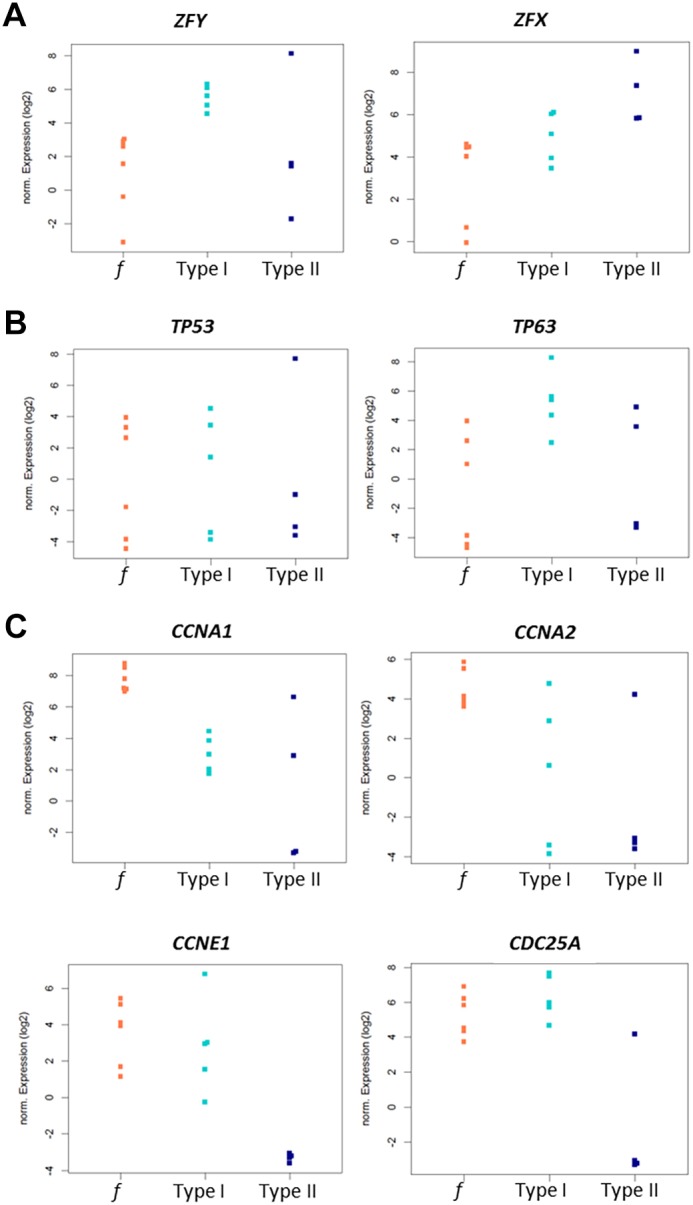
**Patient type-specific expression of genes reflecting aberrant sex chromosome silencing, DNA damage repair and cell cycle regulation.** (A-C) Beeswarm plots depicting different expression levels of *ZFY* (adjusted *P*≤0.0389) and *ZFX* (adjusted *P*≤0.0899) (A), *TP53* (adjusted *P*≤0.8030) and *TP63* (adjusted *P*≤0.0469) (B) and *CCNA1* (adjusted *P*≤0.0051), *CCNA2* (adjusted *P*≤0.0626), *CCNE1* (adjusted *P*≤0.0001) and *CDC25A* (adjusted *P*≤0.0031) (C) in fertile (*f*; orange), type I (turquoise) and type II (dark blue) spermatocytes.

In the mouse (Burgoyne et al., 2009; Jan et al., 2012; Royo et al., 2010), type I meiotic prophase arrest can be caused by incomplete synapsis of the homologous chromosomes and subsequent failure to silence the sex chromosomes, leading to the expression of *Zfy* genes and spermatocyte apoptosis. Human type I arrested spermatocytes also show severe asynapsis of the homologous chromosomes and absence of an XY body (Fig. 2) in which transcription is normally silenced (Fig. S1). We therefore made beeswarm plots to visualize differential expression of the human *ZFY* gene in the spermatocytes of fertile men and men with type I and II spermatocyte arrest. Indeed, we found *ZFY* to be clearly upregulated in type I arrested spermatocytes (Fig. 5A). Hence, also during human male meiosis, asynapsis of the homologous chromosomes may lead to expression of *ZFY* and subsequent spermatocyte elimination. Interestingly, the X chromosome-encoded gene *ZFX*, in the mouse expressed during the interphase between meiosis I and meiosis II (Vernet et al., 2014), is specifically over-expressed in type II arrested spermatocytes (Fig. 5A) and may be thus be involved in the elimination of these cells.

### Type I human arrested spermatocytes display upregulation of the DNA damage response protein p63

Alternatively, type I meiotic arrest could be caused by the DNA damage pathway, involving ATM, CHK2 and the DNA damage response proteins p53 or p63 (Pacheco et al., 2015; Di Giacomo et al., 2005; Li and Schimenti, 2007; Bolcun-Filas et al., 2014). We therefore made additional beeswarm plots to visualize differential expression of the human *TP53* and *TP63* genes (coding for p53 and p63) in fertile men and type I and II patients. The gene *TP53* did not detectably change in type I or II arrested spermatocytes. However, we did find a strong upregulation of *TP63* in type I arrested spermatocytes (Fig. 5B). Hence, DNA damage checkpoint-induced apoptosis of human type I arrested spermatocytes is probably mediated by activation of the DNA damage response protein p63.

### Type II human arrested spermatocytes display lower expression of cell cycle-regulating genes

In the mouse, synapsis and DNA damage checkpoints have been shown to induce meiotic arrest analogous to human type I arrest. Conversely, mouse models showing type II arrest have not been clearly described. However, because disturbed cell cycle regulation seems to be a main cause or consequence of type II arrest, we checked the current literature for mouse knockout models used in cell cycle research. Interestingly, *Ccna1^−/−^* mice, which lack the gene encoding cyclin A1, display a type II-like meiotic prophase arrest without apparent problems at the pachytene stage (Liu et al., 1998; Nickerson et al., 2007). Moreover, in both mouse and human, cyclin A1 is mostly restricted to the testis and in the mouse is present from late pachytene until the meiotic M phases (Wolgemuth et al., 2013). However, in a beeswarm plot, *CCNA1* (the human gene coding for cyclin A1) appears to be downregulated in both type I and type II spermatocytes whereas downregulation of *CCNA2* is more evident in type II cells (Fig. 5C). Also, knockout of cyclin E2, but not cyclin E1, causes meiotic arrest in the mouse (Geng et al., 2003) and together both E-type cyclins control chromosome pairing, telomere stability and CDK2 localization during male meiosis in the mouse (Martinerie et al., 2014). However, in contrast to the mouse, our transcriptome data does not show differential expression of human *CCNE2*, but instead find a clear type II-specific downregulation of *CCNE1*, the gene encoding human cyclin E1 (Fig. 5C). In addition, and also specific for type II arrested spermatocytes, we find a clear downregulation of *CDC25A* (Fig. 5C), a cell cycle-regulating phosphatase expression of which has been found to be significantly decreased in a subgroup of men suffering from meiotic arrest (Cheng et al., 2006).

### Type I and II arrested spermatocytes fail to silence the sex chromosomes properly

**Fig. 6.**
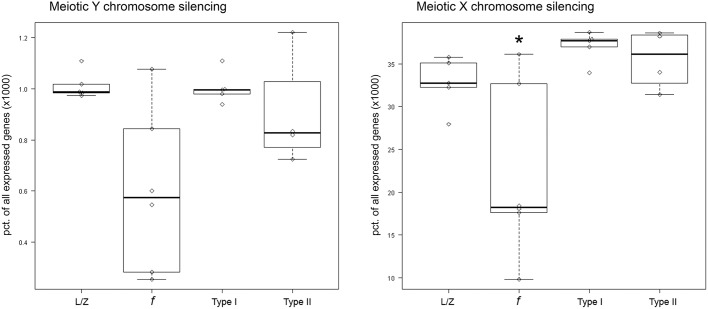
**Aberrant sex chromosome silencing in type I and II arrested spermatocytes.** Boxplots showing the amount of genes expressed from the X and Y chromosomes relative to the total amount of genes expressed in the same sample for leptotene/zygotene (L/Z), fertile control pachytene (*f*), type I arrested and type II arrested spermatocytes. A significant difference was detected between fertile and arrested spermatocytes (two-way ANOVA, Tukey HSD) (*adjusted *P*≤0.0001).

Finally, we investigated whether the increased expression of *ZFY* and *ZFX* in the meiotic arrest samples could be due to disturbed meiotic sex chromosome silencing. We therefore, for each sample, plotted the number of genes expressed from the sex chromosomes relative to the total amount of expressed genes in that specific patient sample, alongside the leptotene/zygotene samples (Fig. 6). Meiotic sex chromosome silencing appeared to be disturbed in both patient groups. However, the relative number of genes expressed from the Y chromosome is very limited and a two-way ANOVA analysis of the two patient groups and controls with normal spermatogenesis, followed by a Tukey's HSD test, only demonstrated significantly disturbed meiotic silencing of the X chromosome (adjusted *P*≤0.0001).

## DISCUSSION

As in mouse (Burgoyne et al., 2009; Jan et al., 2012; Royo et al., 2010), human type I meiotic prophase arrest appears to be characterized by incomplete synapsis of the homologous chromosomes and subsequent failure to silence the sex chromosomes, leading to aberrant expression of the Y-chromosomal gene *ZFY*. However, we did not detect significant patient-specific overall silencing of the Y chromosome. This could be due to the fact that expression of many genes present in human pachytene meiotic cells is already established in early meiotic cells or even in spermatogonia (Jan et al., 2017). Because they are already expressed before chromosome silencing occurs, impaired meiotic silencing of these genes is more difficult to detect. Moreover, because the percentage of genes expressed from the Y chromosome is below 0.1%, differences between patient groups might be too small to detect. The number of genes expressed from the X chromosome is much higher, and indeed we observe a clear increase of X chromosome-expressed genes in both patient groups compared with controls with normal spermatogenesis. As the X and Y chromosomes are always silenced together in the meiotic sex-body, one may assume that failed X chromosome silencing goes together with failed silencing of the Y chromosome. Hence, analogous to the mouse synapsis checkpoint, during human male meiosis, asynapsis of the homologous chromosomes seems to lead to increased expression of *ZFY*, most likely caused by aberrant sex-chromosome silencing.

In addition, meiotic cells can be eliminated during prophase by a separate DNA damage checkpoint involving ATM, CHK2 and the DNA damage response proteins p53 or p63 (Pacheco et al., 2015; Di Giacomo et al., 2005; Li and Schimenti, 2007; Bolcun-Filas et al., 2014; Marcet-Ortega et al., 2017). In line with the presence of such a checkpoint, we find a clear upregulation of *TP63* (but not *TP53*) in human type I arrested spermatocytes. We did not find differential expression of putative upstream regulators of this pathway, for instance MRE11, NBS1, ATM or CHK2, in our dataset. The reason for this result could be that these proteins are regulated by post-translational modifications and remain stable at the transcriptome level. Also in mouse oocytes, which do not contain a Y chromosome or XY silencing, the DNA damage checkpoint leads to specific activation of p63 (Bolcun-Filas et al., 2014). Moreover, it has been recently found that p53 and p63 are specifically involved in recombination-dependent mouse spermatocyte arrest (Marcet-Ortega et al., 2017). We therefore propose that elimination of human type I arrested spermatocytes is likely to be induced by a DNA damage signaling cascade that activates p63.

Although many genetic mouse models display meiotic prophase arrest analogous to the type I human meiotic arrest we describe here, only a few mouse models describe a type II-like meiotic prophase arrest similar to that we describe for type II arrested human spermatocytes. This is probably due to the fact that most genes studied in mouse meiosis are involved in chromosome pairing and synapsis or DSB repair, processes that when disturbed will mostly lead to type I-like arrest (De Rooij and De Boer, 2003; Hamer et al., 2008; Pacheco et al., 2015). In contrast, we selected our patient samples based on testicular histology and were thus unbiased with respect to their genetic background. From our data, it appears that the type II meiotic prophase arrest we describe for human meiosis can be defined by a decrease in transcripts required for cell cycle progression, especially the cyclins A2 and E1 and the cell cycle-regulating phosphatase *CDC25A*. Indeed, in line with these data, disruption of cyclins in mice can lead to type II-like meiotic arrest (Liu et al., 1998; Nickerson et al., 2007; Martinerie et al., 2014). The exact mechanism that underlies type II arrested spermatocyte elimination remains to be elucidated. Considering the differential expression of genes involved in chromosome organization and RNA processing in both types of human meiotic prophase arrest, these processes could form an underlying problem for prophase meiotic arrest in general. However, in type II arrested spermatocytes these problems may be more subtle and, in contrast to type I, not directly apparent at the microscopy level. Such less apparent problems, although not detected by the genome integrity checkpoints that induce expression of *ZFY* or *TP63* in type I arrested spermatocytes, may still induce cell cycle arrest and subsequent type II meiotic failure. On the other hand, we have now demonstrated that, despite apparently normal chromosome synapsis and XY-body formation, type II arrested spermatocytes also display disturbed sex chromosome silencing. However, in type II arrested spermatocytes this seems to lead to increased expression of the X chromosome-encoded gene *ZFX*, suggesting a separate elimination pathway that is more active in type II arrested spermatocytes. Interestingly, in type II arrested spermatocytes, X-chromosome silencing was also clearly disturbed at the transcriptome level, even when cytology of the XY body seemed to be unaffected.

One could argue that spermatocytes in type I and II meiotic arrest patients simply do not progress far enough to get the chance to express genes that are usually present in healthy pachytene spermatocytes. Importantly, we used the transcriptome of early pachytene spermatocytes, collected in a previous study describing gene expression throughout normal spermatogenesis (Jan et al., 2017), as controls. In this study, almost all genes characteristic for meiosis appeared to be already expressed in early spermatocytes, with early and late spermatocytes only displaying 24 DEGs. Of these 24 genes, only four, which were all downregulated in late pachytene spermatocytes in the previous study, were also found as differentially regulated in the current study. Therefore, differences in gene expression found in the arrested spermatocytes are not likely to be due to failure to reach the later pachytene-like stage at which these genes would normally start to be expressed.

Spermatocytes of one patient, initially classified as type II, appeared to display a transcriptome profile similar to that of pachytene spermatocytes from fertile men. Like type II arrest, arrested spermatocytes from this patient showed normal chromosome behavior and crossover formation, with the only difference being the presence of occasional seminiferous tubules containing metaphase spermatocytes. Meiosis of this patient thus had no problems during the meiotic prophase but instead arrested at a meiotic metaphase stage. Considering the huge genetic and phenotypic diversity among human patients, it is not possible to delineate a list of statistically significant genes that would describe a common denominator for human male meiotic metaphase arrest from only a single patient. We therefore focused on the two types of human meiotic prophase arrest of which we analyzed the arrested spermatocytes of five and four patients, respectively.

Our study presents a comprehensive and publicly available list of genes and pathways that are involved in two types of human meiotic prophase arrest. Identification and understanding of these meiotic arrest mechanisms increases our insight into how genomic stability is guarded during human germ cell development.

## MATERIALS AND METHODS

### Tissue collection

Testicular biopsies were collected with informed consent from men with non-obstructive azoospermia undergoing a testicular sperm extraction procedure (TESE) for potential intracytoplasmic sperm injection treatment at the Center for Reproductive Medicine at Amsterdam Medical Centrum (AMC). All men were diagnosed with idiopathic non-obstructive azoospermia as part of routine fertility work-up after failure to conceive naturally after at least one year of unprotected sexual intercourse with their partner. After the diagnosis of non-obstructive azoospermia was made, all men were karyotyped and screened for deletions of the Y chromosome (*AZFa*, *AZFb*, *AZFc* and *gr/gr* deletions). All men had a normal 46, XY karyotype and did not have any Y-chromosome deletions. As controls in our bioinformatics analysis, we included vasectomy reversal patients with full spermatogenesis (judged on histological testis sections), who had fathered children before vasectomy [see Jan et al. (2017) for a full description]. For histology and cytology, we used testis sections from a patient with obstructive azoospermia, showing complete spermatogenesis in all the seminiferous tubules. Biopsies were fixed in modified methacarn (89% methanol and 11% glacial acetic acid) and embedded in paraffin as described previously (Jan et al., 2017) and later used for laser capture microdissection. Remnants of the TESE procedure after sperm extraction were cryopreserved in 8% dimethyl sulfoxide (DMSO, Sigma-Aldrich) and 20% fetal calf serum (FCS) (Invitrogen) in minimum essential medium (MEM, Invitrogen) and stored at −196°C for later use in preparing meiotic spreads. Patient IDs included in the study were: AMC1805, AMC2281, AMC2489, AMC2188, URO0225, URO0229, URO0287, AMC2196, AMC2226 and AMC2062 and, for histology and cytology, AMC1849.

### Immunochemistry

Immunohistochemical staining with mouse monoclonal anti-γH2AX (1:20,000; 05-636, Millipore), on 5-µm-thick human testis sections, was performed as described previously (Verver et al., 2013, 2014). Human meiotic spread preparations were prepared according to an adapted protocol from De Vries et al. (2012). Briefly, germ cells were isolated from testicular TESE remnants using enzymatic digestion with collagenase IV in Hank's balanced salt solution (HBBS; Gibco)/DNase solution for 20 min at 37°C. Subsequently, loosened tubules were incubated in a solution containing 0.25% trypsin/EDTA diluted 1:5 in HBBS/DNase at 37°C until the biopsies were completely dissociated. Trypsin was inactivated using 5 ml of MEM/10% FCS. Following this, the dissociated tissue was spun down at 350 ***g*** for 5 min without break. The supernatant was removed and the resulting pellet was re-suspended in testis cell isolation medium [104 mM NaCl, 45 mM KCl, 1.2 mM MgSO_4_, 0.6 mM KH_2_PO_4_, 0,1% (w/v) glucose, 6 mM sodium lactate, 1 mM sodium pyruvate; pH adjusted to 7.3 with HCl and filter-sterilized]. Hereafter, for spreading of the cells, the meiotic spreads protocol from De Vries et al. (2012) was followed. For immunocytological staining, spreads were blocked in 1% FCS in PBS for 30 min at room temperature followed by an overnight incubation with the following primary antibodies: mouse anti-γH2AX (1:20,000; Millipore) and goat anti-SYCP3 (1:500; AF3750, R&D Systems). The spreads were subsequently incubated for 1 h with secondary antibodies: Alexa 555-conjugated donkey anti-mouse and Alexa 488-conjugated donkey anti-goat (A-31570 and A-11055, Invitrogen). Slides were then counterstained with DAPI and mounted using ProLong Gold (Cell Signaling Technology). For MLH1 staining, mouse anti-MLH1 (1:500; 554073, BD Pharmingen) and goat anti-SYCP3 (1:500; R&D Systems) were used with their respective secondary antibodies Alexa 488-conjugated donkey anti-mouse and Alexa 555-conjugated donkey anti-goat (A-21202 and A-21432, Invitrogen).

### Cot-1 DNA fluorescence *in situ* hybridization (FISH)

To visualize nascent RNA sequences and proteins in the same sample, meiotic spread slides were first subjected to the standard immunofluorescence protocol as described above, after which they underwent a Cot-1 RNA-FISH protocol. Human Cot-1 DNA (Roche) was biotin labeled by nick translation and used as a probe, diluted in a 50% formamide hybridization mix adapted from Turner et al. (2005) [50% formamide, 4× sodium chloride/sodium citrate solution (SSC), 20% dextran sulfate, 2 mg/ml DNase/RNase-free bovine serum albumin (BSA)]. Probe solution was denatured at 72°C for 10 min, chilled on ice and added to the meiotic spreads slides for an overnight incubation at 37°C. The following morning slides were washed three times in 1× SSC/50% formamide solution at 42°C followed by three washes with 2× SSC at 42°C and one rinse with 4× SSC+0.1% Tween20 at room temperature. The slides were subsequently blocked in FISH blocking solution (4× SSC, 0.1% Tween 20, 4 mg/ml DNase/RNase-free BSA for 30 min at 37°C. Slides were then incubated with avidin-Cy3 (1:5000; 200-162-211, Jackson ImmunoResearch). To enhance the signal, slides were then incubated with biotinylated anti-avidin (1:500; BA-0300, Vector Laboratories) followed by a final incubation with avidin-Cy3; each incubation was for 30 min at 37°C. Finally, the slides were stained for yH2AX and SCP3 as described above.

### Microscopy

Bright-field microscopy images were acquired at room temperature using an Olympus BX41 microscope equipped with an Olympus DP20 color camera. Fluorescence microscopy images were acquired at room temperature using a Plan Fluotar 100×/1.30 oil objective on a Leica DM5000B microscope equipped with a Leica DFC365 FX CCD camera. Images were analyzed using Leica Application Suite Advanced Fluorescence (LAS AF) software. The figures were constructed using Adobe Photoshop CS5 version 12.0.

### Single-cell laser capture microdissection and RNA preparation

Directly prior to laser dissection microscopy (LDM), 5 µm-thick sections of testis tissue were mounted on Superfrost glass microscope slides (Thermo Fisher Scientific) and stained with Hematoxylin and Eosin as described previously (Jan et al., 2017). For each patient, 500 histologically pachytene-like spermatocytes were individually laser dissected and pooled as described previously (Jan et al., 2017). Laser-dissected cells were captured in silicon-coated adhesive caps (Adhesive cap 500 opaque tube, Zeiss) and were lysed at 42°C in 10 µl of extraction buffer provided in the PicoPure RNA isolation kit (Arcturus). Cell lysates were stored at −80°C until further use. All procedures were performed under RNase-free conditions. Total RNA was isolated from cell lysates using the PicoPure RNA isolation kit (Arcturus) according to the manufacturer's protocol, including an on-column DNase treatment. RNA was eluted in 10 µl elution buffer. Subsequently, the RNA was concentrated to a volume of 5 µl with a speed vacuum centrifuge for 8 min. Total RNA isolated from 500 pooled cells was SPIA-amplified using the Ovation RNAseq V2 System (Nugen) as described previously (Jan et al., 2017).

### RNA sequencing

The amplified cDNA was sheared using the Covaris S220 (Thermo Fisher Scientific). DNA libraries were made from the SPIA-amplified cDNA and sequenced single-end, 50 bps on the HiSeq2000 Illumina platform obtaining at least 10 million reads using 8 pmol per library.

### Bioinformatics

Bioinformatics analysis was carried out using a pipeline previously described (Jan et al., 2017). Briefly, samples with a normalization factor between 0.6 and 1.4 were included in the analyses. For multidimensional scaling analysis, comparing previously derived germ cell transcriptomes with arrested type I or II spermatocytes (Jan et al., 2017), all samples included in the plot were normalized together. For all further analyses, the leptotene/zygotene, pachytene and type I and II arrested spermatocytes were normalized together. A gene was considered to be expressed if it had >1 count per million present in at least three individuals per sample group. Raw counts were transformed to moderated log counts per million before filtering using the cpm function with default parameters. A list of DEGs between the spermatocytes was obtained by estimating the mean variance of the log counts using the voom method and analyzing these with the empirical Bayes pipeline as implemented in limma (version 3.22.7). After correcting for multiple testing, a *P*-value of <0.05 was considered significant for DEG analysis. K-means clustering (default algorithm) was used to obtain plots for the scaled normalized relative gene expression data on a log scale for each expressed gene using packages clValid (version 0.6-6), cluster (version 2.0.2) and stats. Gene ontology analysis was performed using the functional annotation clustering tool in DAVID. An enrichment score of >1.3 was considered significant.
